# Supplementary material for: Internal and external protective factors associated with the secondary traumatic stress component of compassion fatigue, in feral cat caregivers
Source: PLoS One. 2026 Aug 3;21(8):e0354464. doi: 10.1371/journal.pone.0354464 (PMC13432124; doi:10.1371/journal.pone.0354464)
Supplement: S1 File — (PDF) [file pone.0354464.s001.pdf]

S1 File. English translation of ethics approval

(Translation of original Portuguese document)

Report

332/CEFMUP/2025

Ethics Committee of the Faculty of Medicine of the University of Porto

Report: 332/CEFMUP/2025

Project title: Internal and External Factors that Protect Caregivers of Feral Cats from Compassion Fatigue

Researcher: Cristina Maria Nogueira da Costa Santos

Report:

☑ Favourable / Accepted

☐ Rejected / Declined

☐ Other

Rapporteur: Prof. Dr. Patrícia Ribeiro

Deliberated in plenary meeting of the Ethics Committee of Porto, on March 25, 2025, by unanimity of the members present.

It is kindly requested that, after the conclusion of the project, the final report with the conclusions of the study be submitted, in accordance with Article 3, no. 3, paragraph f) of Decree-Law no. 80/2018 of October 15.

Porto, March 25, 2025

The Secretariat of the Ethics Committee

Prof. Dr. Francisca Rego

The President of the Ethics Committee

Prof. Dr. Rui Nunes
