# Supplementary material for: Internal and external protective factors associated with the secondary traumatic stress component of compassion fatigue, in feral cat caregivers
Source: PLoS One. 2026 Aug 3;21(8):e0354464. doi: 10.1371/journal.pone.0354464 (PMC13432124; doi:10.1371/journal.pone.0354464)
Supplement: Attachment — Submitted filename: Answer.docx [file pone.0354464.s003.docx]

Dear Editors and Reviewers

We sincerely thank the Editor and the Reviewers for their thorough evaluation of our manuscript and for their valuable and constructive comments. Their thoughtful suggestions have enabled us to improve the manuscript substantially in terms of clarity, methodological transparency, and scientific rigor. We have carefully considered all comments and revised the manuscript accordingly. Below, we provide a detailed point-by-point response to each of the issues raised.

**Review Comments to the Author**

**Reviewer #1:**

*(...) The manuscript is generally well written, particularly the Introduction section, which clearly contextualizes the problem and the rationale of the study. In addition, the Materials and Methods section is well described and easy to follow. In this section, it would be useful to specify the score ranges used to determine the levels of each questionnaire (e.g., low, moderate, or high levels). This would allow readers to interpret the results more easily and understand which scores correspond to “moderate” or “high” levels.*

**Answer:**

We thank the reviewer for this helpful comment. As suggested, we have added to the Methods section the cut-off values and score ranges used to classify participants into low, moderate, and high levels for the different questionnaires.

*I would also strongly recommend including additional summary tables to help readers better understand the data presented. In the Results section (page 9, line 224: “mostly women [164; 94%]”), I suggest including a summary table presenting the descriptive statistics for all questionnaire results, including means and standard deviations. The authors could also consider including medians and ranges (minimum–maximum values), if appropriate. For example, in line 256 the authors state that “most participants reported high perceived social support,” but no supporting numerical data are provided.*

**Answer:**

Following this recommendation, we have included two additional summary tables presenting the descriptive statistics for sociodemographic characteristics, feral cat colony characteristics and psychosocial measures. We have also incorporated the other suggested descriptive information in the text to facilitate the interpretation of the data and provide readers with a clearer overview of the study sample and questionnaire results.

*Regarding Figure 1, the presentation is somewhat confusing because percentages are not shown relative to the total study population, which makes interpretation more difficult.*

**Answer:**

We thank the reviewer for this valuable comment. Following this suggestion, Figure 1 was revised to include both the absolute number and the percentage of participants in each district. This modification facilitates comparison between the sample distribution and the population distribution across districts.

*There are also some minor language issues. In the tables, the expression “our mais” should probably read “or more.” Additionally, there appears to be a grammatical error in line 314.*

**Answer:**

We thank the reviewer for this observation. The manuscript has been carefully revised, and the grammatical and typographical errors identified by the reviewer have been corrected throughout the text.

*Importantly, the statement in lines 316–318 requires an appropriate reference citation. More generally, the Discussion section lacks sufficient references and does not adequately compare the present findings with previous literature. I believe this entire section should be substantially revised and expanded.
Specifically, the authors should include references to previous studies on secondary traumatic stress and compassion fatigue in individuals working with animals, including veterinarians, shelter volunteers, animal rescue workers, and related professionals. There is currently a considerable body of literature on this topic, including recent studies identifying social support as a protective factor against compassion fatigue in animal-care professions. Including studies from Portugal and other countries would further strengthen the international relevance of the discussion.*

**Answer:** We thank the reviewer for this valuable comment. In response, we have substantially revised and expanded the Discussion section. Additional references were included to support the statements made, and our findings are now discussed in relation to previous studies on compassion fatigue.

**Reviewer #2:**

*Line 15: I think there’s a bit of a leap here between professionals with codes of conduct and many pressures and informal caregivers of cat colonies. Generally, informal caregivers are under-researched, let alone cat colony caregivers*

**Answer:** We thank the reviewer for this comment. We agree that important differences exist between healthcare professionals and informal feral cat caregivers, particularly regarding professional responsibilities, codes of conduct, and organizational contexts. We have revised the manuscript to acknowledge these distinctions more explicitly. However, we believe that comparisons with healthcare professionals remain relevant because both groups are exposed on a daily basis to suffering, death, ethical dilemmas, and emotionally demanding caregiving situations, which are key factors associated with compassion fatigue. We also emphasize that feral cat caregivers often operate with limited resources and support, which may represent a distinct source of stress in this population.

*Line 42 – Is compassion stress inevitable?*

**Answer:** We agree with the reviewer that the term "inevitable" was too strong and could imply that compassion stress is unavoidable. Accordingly, we have removed this term from the manuscript.

*Line 74: The veterinary professional (no s)*

**Answer:** We thank the reviewer for identifying this error. The manuscript has been corrected accordingly.

*Line 130: Provide reference 22 earlier, e.g. Resilience can be divided into four factors [22]: Perserverance…
Line 139: Likewise, provide reference 23 earlier, Spiritual well-being can be defined [23] as a dynamic*

**Answer:** The manuscript has been corrected accordingly.

*Line 139: I find this interesting, but I’m not sure I see the relevance here. Is it because this population is strongly spiritual? Can you make the relevance clearer to the reader?*

**Answer:** We thank the reviewer for this observation. To clarify the relevance of this finding, we added references to previous studies in nurses that identified spiritual well-being as a protective factor against compassion fatigue and subsequently discussed how these findings compare with our results in feral cat caregivers.

*Line 191 & Line 204: can you define these psychometric properties and what makes them good? E.g. do they show good coherence?*

**Answer:** We thank the reviewer for this comment. The concepts of resilience, spiritual well-being, and perceived social support are defined in the Introduction section. To further clarify their relevance, we have also added a brief explanation in the Methods section stating that these variables were included because previous research identified them as potential protective factors against compassion fatigue and psychological distress.

*Line 218/Line285: It seems you have taken a content analysis style approach (or reliable thematic analysis) to the free text data. This needs to be outlined alongside your researcher positionality somewhere in the methods.*

**Answer:** We thank the reviewer for this comment. We have added a description of the qualitative analysis procedure to the Methods section. One of the authors read all open-ended responses and conducted an inductive content analysis, whereby categories were developed iteratively from the data. The responses were then coded according to these categories and analyzed descriptively. As the qualitative component was exploratory, not included in regression models and intended to complement the quantitative findings, no formal thematic analysis framework was applied.

*Line 224: ‘mostly women’ – specify how many, and give measure of variance like SD alongside mean age Similarly, provide percentage for statements like ‘majority were employed’.*

**Answer:** We agree with the reviewer's comment. The text has been revised to specify the exact numbers and percentages. Furthermore, two new tables have been added to the manuscript to better clarify and present these descriptive statistics.

*Line 226: This reads as though you have a single estimate of colony size for each respondent, which you later say you don’t. Can you be clearer as to how this was asked.*

**Answer:** We thank the reviewer for this comment. Colony size was assessed by asking participants to select the category that best represented the approximate number of cats they cared for on a daily basis. To address the reviewer’s concern, we have revised the manuscript to clarify this procedure in the Methods section and have also improved the presentation of the corresponding results.

*Line 230: Does it always follow that a larger colony is a higher caregiving burden? Presumably you don’t necessarily look after a large colony alone? (Even if you don’t engage with other caregivers they could still be supporting your colony?)*

**Answer:** We have revised the manuscript and removed the interpretation that larger colony size represents a higher caregiving burden.

*Line 232: Whose benchmarks are you using for your ‘acceptable’ and ‘good’ thresholds of Cronach’s alpha?*

**Answer:** We thank the reviewer for this comment. We have clarified the criteria used to interpret Cronbach’s alpha coefficients in the Methods section. Following commonly used guidelines, values ≥0.70 were considered acceptable and ≥0.80 good. (1).

(1) George, D., & Mallery, P. (2003). SPSS for Windows Step by Step: A Simple Guide and Reference (4th ed.). Allyn & Bacon.

*Line 242: Similarly, I would like reported mean, median, and ranges for your scores, and a better understanding of the thresholds for ‘high’ and ‘moderate’ etc. Some of this could be incorporated into figures.*

**Answer:** We thank the reviewer for this suggestion. In the revised manuscript, we added summary tables presenting the descriptive statistics of all questionnaire and included the score ranges used to classify the levels of each questionnaire, thereby facilitating the interpretation of the results.

*Lines 261-284: You report the univariate and the multivariate models. Surely the univariate models are actually part of your overall model selection process? What value do you see in reporting them separately, because the moment that’s not clear to me, especially as they’re not narratively reported in the text. With regards to the multivariate model, why does your final model retain measures with no impact on the dependent variable? Is it not more parsimonious to remove those so you’re not spending degrees of freedom on modelling them? Prior to reporting the table, I would like a narrative report of the model (Younger, unemployed caregivers of colonies with more than 25 cats, lower Resilience scores, and lower family social support scores were more likely to have higher Secondary Traumatic Stress scoring) – you might find the ‘report’ package in R useful here (it’s one of my favourites) for actually giving us the effect size of each of these parameters which should be reported more clearly.*

**Answer:** We thank the reviewer for this thoughtful comment. The univariate models were included because, as described in the Methods section, variables with a p-value < 0.20 in the univariate analyses were selected for inclusion in the multivariable model. Therefore, reporting all univariate coefficients and p-values is important to provide transparency regarding the variable selection process and to allow readers to understand how the final adjusted model was specified.

Regarding the multivariable model, we chose to adjust for all variables meeting the predefined inclusion criterion (p < 0.20 in the univariate analyses), regardless of whether they remained statistically significant after adjustment. This approach was adopted to ensure adequate control for potential confounding and to evaluate the independent contribution of each variable while accounting for the others. Standardized regression coefficients are reported, allowing readers to compare the relative importance of the different predictors of Secondary Traumatic Stress.

*Line 362 – The link between spiritual well being and this study still isn’t cl;ear to me*

**Answer:** We thank the reviewer for this comment. In the revised version of the manuscript, we have clarified the rationale for including spiritual well-being in this study. Previous studies conducted among nurses identified spiritual well-being as a protective factor against compassion fatigue. Therefore, we sought to examine whether a similar protective effect could be observed among informal feral cat caregivers. However, unlike findings reported in healthcare professionals, spiritual well-being was not independently associated with Secondary Traumatic Stress in our study. This rationale and the comparison with previous literature are now more clearly explained in both the Introduction and Discussion sections.

*Line 367 – Requiring a telephone number likely excludes unhoused people? (Maybe that’s old fashioned of me)*

**Answer:** We thank the reviewer for this observation. We agree that requiring a telephone number may have excluded some potential participants, including individuals experiencing homelessness. Although we cannot rule out this possibility, we believe that its impact on the study sample is likely to be limited, as the daily care of feral cat colonies typically requires sustained logistical organization, regular access to resources, and a long-term commitment. These requirements may pose additional challenges for individuals experiencing homelessness. Moreover, access to a telephone was essential to ensure nationwide coverage of the study.

.

*Line 396 – really interesting*

**Answer:** We thank the reviewer for highlighting this finding. Beyond its relevance for understanding the factors associated with Secondary Traumatic Stress, it may also have practical implications for municipal policies regarding feral cat colony management. The absence of a protective effect associated with formal colony registration or contact with municipal veterinary services suggests that current forms of institutional support may not adequately address the emotional and practical needs of caregivers. This finding may point to the need for alternative or more comprehensive municipal support strategies for informal feral cat caregivers.

*Stylistic note*

*I felt some of your paragraphs could do with more connections. We start about compassion fatigue, then jump to cat natural history, then start talking about one health approaches. At about Line 77 I would advise you to talk about one health and one welfare concepts, highlighting the relationship between people and animals, and then note that caregiver fatigue may extend beyond professionals caring for animals, but also to informal caregivers, and that this is relatively under-researched. You can then highlight cat colonies as an example of this, give the history of cat colonies and cats, and then move on to what you have in line 127ish*

**Answer:** We thank the reviewer for this helpful suggestion. We agree that some sections of the Introduction could benefit from stronger transitions and a clearer logical flow. In the revised manuscript, we introduced some modifications to improve the connections between the different components of the Introduction. We believe these changes have resulted in a more coherent and logically structured narrative.

**Journal Requirements:**

***1.*** *Please ensure that your manuscript meets PLOS ONE's style requirements, including those for file naming. The PLOS ONE style templates can be found at*

[*https://journals.plos.org/plosone/s/file?id=wjVg/PLOSOne_formatting_sample_main_body.pdf*](https://journals.plos.org/plosone/s/file?id=wjVg/PLOSOne_formatting_sample_main_body.pdf) *and* 
[*https://journals.plos.org/plosone/s/file?id=ba62/PLOSOne_formatting_sample_title_authors_affiliations.pdf*](https://journals.plos.org/plosone/s/file?id=ba62/PLOSOne_formatting_sample_title_authors_affiliations.pdf)

**Answer:**

The manuscript has been reviewed and revised to ensure compliance with all relevant PLOS ONE formatting and style requirements.

***2.*** *In the online submission form you indicate that your data is not available for proprietary reasons and have provided a contact point for accessing this data. Please note that your current contact point is a co-author on this manuscript. According to our Data Policy, the contact point must not be an author on the manuscript and must be an institutional contact, ideally not an individual. Please revise your data statement to a non-author institutional point of contact, such as a data access or ethics committee, and send this to us via return email. Please also include contact information for the third party organization, and please include the full citation of where the data can be found.*

**Answer:**

We have revised the Data Availability Statement accordingly and replaced the author contact with an institutional contact point. The revised Data Availability Statement has been returned by email.

The revised statement reads:

"Data are not publicly available due to ethical and privacy restrictions. Requests for access to anonymized data should be submitted by email to the Department of Community Medicine, Information and Health Decision Sciences, Faculty of Medicine of the University of Porto, Portugal, at [medcids@med.up.pt](mailto:medcids@med.up.pt) (data reference: **Component of compassion fatigue, in feral cat caregivers – Cristina Costa Santos**). Access to the data is subject to **approval by the relevant ethics and data protection authorities."**

***3.*** *We note that Figure 1 in your submission contain map images which may be copyrighted. All PLOS content is published under the Creative Commons Attribution License (CC BY 4.0), which means that the manuscript, images, and Supporting Information files will be freely available online, and any third party is permitted to access, download, copy, distribute, and use these materials in any way, even commercially, with proper attribution. For these reasons, we cannot publish previously copyrighted maps or satellite images created using proprietary data, such as Google software (Google Maps, Street View, and Earth). For more information, see our copyright guidelines:* [*http://journals.plos.org/plosone/s/licenses-and-copyright*](http://journals.plos.org/plosone/s/licenses-and-copyright)*.
 
We require you to either (1) present written permission from the copyright holder to publish these figures specifically under the CC BY 4.0 license, or (2) remove the figures from your submission:
 
1) You may seek permission from the original copyright holder of Figure 1 to publish the content specifically under the CC BY 4.0 license.  
 
We recommend that you contact the original copyright holder with the Content Permission Form (*[*http://journals.plos.org/plosone/s/file?id=7c09/content-permission-form.pdf*](http://journals.plos.org/plosone/s/file?id=7c09/content-permission-form.pdf)*) and the following text:
“I request permission for the open-access journal PLOS ONE to publish XXX under the Creative Commons Attribution License (CCAL) CC BY 4.0 (*[*http://creativecommons.org/licenses/by/4.0/*](http://creativecommons.org/licenses/by/4.0/)*). Please be aware that this license allows unrestricted use and distribution, even commercially, by third parties. Please reply and provide explicit written permission to publish XXX under a CC BY license and complete the attached form.”
 
Please upload the completed Content Permission Form or other proof of granted permissions as an ""Other"" file with your submission.
 
In the figure caption of the copyrighted figure, please include the following text: “Reprinted from [ref] under a CC BY license, with permission from [name of publisher], original copyright [original copyright year].”
 
2) If you are unable to obtain permission from the original copyright holder to publish these figures under the CC BY 4.0 license or if the copyright holder’s requirements are incompatible with the CC BY 4.0 license, please either i) remove the figure or ii) supply a replacement figure that complies with the CC BY 4.0 license. Please check copyright information on all replacement figures and update the figure caption with source information. If applicable, please specify in the figure caption text when a figure is similar but not identical to the original image and is therefore for illustrative purposes only.*

*The following resources for replacing copyrighted map figures may be helpful:
 
USGS National Map Viewer (public domain):* [*http://viewer.nationalmap.gov/viewer/*](http://viewer.nationalmap.gov/viewer/) *The Gateway to Astronaut Photography of Earth (public domain):* [*http://eol.jsc.nasa.gov/sseop/clickmap/*](http://eol.jsc.nasa.gov/sseop/clickmap/) *Maps at the CIA (public domain):* [*https://www.cia.gov/library/publications/the-world-factbook/index.html*](https://www.cia.gov/library/publications/the-world-factbook/index.html) *and* [*https://www.cia.gov/library/publications/cia-maps-publications/index.html*](https://www.cia.gov/library/publications/cia-maps-publications/index.html) *NASA Earth Observatory (public domain):* [*http://earthobservatory.nasa.gov/*](http://earthobservatory.nasa.gov/) *Landsat:* [*http://landsat.visibleearth.nasa.gov/*](http://landsat.visibleearth.nasa.gov/) *USGS EROS (Earth Resources Observatory and Science (EROS) Center) (public domain):* [*http://eros.usgs.gov/#*](http://eros.usgs.gov/) *Natural Earth (public domain):* [*http://www.naturalearthdata.com/*](http://www.naturalearthdata.com/)

**Answer:**

Thank you for raising this issue.

Figure 1 is an original figure created by the authors in R and does not contain any Google Maps, Google Earth, satellite imagery, or other proprietary map content.

The map was generated using administrative boundary data from the Carta Administrativa Oficial de Portugal (CAOP), the official administrative boundary dataset of Portugal maintained and distributed by the Portuguese government through the Directorate-General for Territory (DGT) - https://www.dgterritorio.gov.pt/atividades/cartografia/cartografia-tematica/caop. The CAOP dataset is distributed under the Creative Commons Attribution 4.0 (CC BY 4.0) licence, which is compatible with the licensing requirements of PLOS ONE.

Therefore, Figure 1 is not based on copyrighted proprietary map content and no additional permission is required for publication under the CC BY 4.0 licence.

To clarify this in the manuscript, we have revised the figure legend to acknowledge the source of the administrative boundaries used in the map.

Thank you for bringing this to our attention.

***4.*** *Please include captions for your Supporting Information files at the end of your manuscript, and update any in-text citations to match accordingly. Please see our Supporting Information guidelines for more information:* [*http://journals.plos.org/plosone/s/supporting-information*](http://journals.plos.org/plosone/s/supporting-information)*.*

**Answer:**

No Supporting Information files were submitted with this manuscript.

***5.*** *If the reviewer comments include a recommendation to cite specific previously published works, please review and evaluate these publications to determine whether they are relevant and should be cited. There is no requirement to cite these works unless the editor has indicated otherwise.*

**Answer:**

Thank you for this guidance. The reviewers did not recommend any additional references for citation.
